# Dynamic Changes of Active Components and Volatile Organic Compounds in *Rosa roxburghii* Fruit during the Process of Maturity

**DOI:** 10.3390/foods13182893

**Published:** 2024-09-12

**Authors:** Su Xu, Junyi Deng, Siyao Wu, Qiang Fei, Dong Lin, Haijiang Chen, Guangcan Tao, Lingshuai Meng, Yan Hu, Fengwei Ma

**Affiliations:** College of Food Science and Engineering, Guizhou Engineering Research Center for Characteristic Flavor Perception and Quality Control of Dual-Food Homologous Resources, Guiyang University, Guiyang 550005, China; xs8515@126.com (S.X.); 13187337873@163.com (J.D.); ceci41@126.com (S.W.); fqorganic@163.com (Q.F.); gyulindong@163.com (D.L.); b05chenhj@126.com (H.C.); tgcan@126.com (G.T.); 15040260380@163.com (L.M.); huyanswu@163.com (Y.H.)

**Keywords:** *Rosa roxburghii*, active components, volatile organic compounds, maturity stage, HS-SPME-GC-MS

## Abstract

*Rosa roxburghii (R. roxburghii*), native to the southwest provinces of China, is a fruit crop of important economic value in Guizhou Province. However, the changes in fruit quality and flavor during *R. roxburghii* fruit ripening have remained unknown. Here, this study investigated the changes of seven active components and volatile organic compounds (VOCs) during the ripening of the *R. roxburghii* fruit at five different ripening stages including 45, 65, 75, 90, and 105 days after anthesis. The results indicated that during the ripening process, the levels of total acid, vitamin C, and soluble sugar significantly increased (*p* < 0.05), while the levels of total flavonoids, superoxide dismutase (SOD), and soluble tannin significantly decreased (*p* < 0.05). Additionally, the content of total phenol exhibited a trend of first decreasing significantly and then increasing significantly (*p* < 0.05). A total of 145 VOCs were detected by HS-SPME-GC-MS at five mature stages, primarily consisting of aldehydes, alcohols, esters, and alkenes. As *R. roxburghii* matured, both the diversity and total quantity of VOCs in the fruit increased, with a notable rise in the contents of acids, ketones, and alkenes. By calculating the ROAV values of these VOCs, 53 key substances were identified, which included aromas such as fruit, citrus, green, caramel, grass, flower, sweet, soap, wood, and fat notes. The aromas of citrus, caramel, sweet, and wood were predominantly concentrated in the later stages of *R. roxburghii* fruit ripening. Cluster heatmap analysis revealed distinct distribution patterns of VOCs across five different maturity stages, serving as characteristic chemical fingerprints for each stage. Notably, stages IV and V were primarily characterized by a dominance of alkenes. OPLS-DA analysis categorized the ripening process of *R. roxburghii* fruit into three segments: the first segment encompassed the initial three stages (I, II, and III), the second segment corresponded to the fourth stage (IV), and the third segment pertained to the fifth stage (V). Following the variable importance in projection (VIP) > 1 criterion, a total of 30 key differential VOCs were identified across the five stages, predominantly comprising ester compounds, which significantly influenced the aroma profiles of *R. roxburghii* fruit. By integrating the VIP > 1 and ROAV > 1 criteria, 21 differential VOCs were further identified as key contributors to the aroma changes in *R. roxburghii* fruit during the ripening process. This study provided data on the changes in quality and aroma of *R. roxburghii* fruit during ripening and laid the foundation for the investigation of the mechanism of compound accumulation during ripening.

## 1. Introduction

*Rosa roxburghii* (*R. roxburghii*) is a valuable wild fruit tree belonging to the Rosaceae family. It is a deciduous bushy shrub that is endemic to China and is commercially grown and consumed in the country. The *R. roxburghii* fruit is widely cultivated in the southwestern provinces of China, particularly in Guizhou Province [1]. Known for its medicinal and edible properties, modern research has highlighted the nutritional and medicinal benefits of *R. roxburghii* fruit [2,3,4]. It is a rich source of vitamin C, superoxide dismutase (SOD), flavonoids, and other active compounds [3,5], showcasing various health benefits such as antibacterial, anti-aging, anti-radiation, and anti-inflammatory properties, and even the ability to inhibit cancer cell proliferation [2,4,6]. These attributes have positioned it as a valuable raw material for the development of high-quality functional foods [6,7].

Flavor, defined as a combination of non-volatile taste and volatile aroma, is the most significant attribute of *R. roxburghii* fruit [8]. Alongside its nutritional and medicinal benefits, the flavor of *R. roxburghii* plays a crucial role in driving consumer interest. Factors such as soluble sugars, vitamin C, total acid, total phenol, tannin, and other active ingredients contribute to the sweet, sour, and astringent taste of the fruit [9,10,11,12]. Additionally, the formation of a strong aroma is linked to volatile organic compounds (VOCs) released by the fruit [13]. The headspace solid-phase micro-extraction gas chromatography–mass spectrometry (HS-SPME-GC-MS) method is widely utilized for the identification and quantification of flavor components in fruits. This method offers the benefits of convenience and speed by automating the solid-phase micro-extraction (SPME) process for flavor enrichment [14,15]. Current research on the aroma of *R. roxburghii* has primarily focused on the analysis of *R. roxburghii* juice. For instance, in a recent study, 37 compounds in *R. roxburghii* juice were identified and quantified using gas chromatography–olfactometry (GC-O) and gas chromatography–mass spectrometry (GC-MS). Among these compounds, ethyl-2-methylpropionate, ethyl butanoate, ethyl-2-methylbutanoate, and ethyl hexanoate were found to have higher odor activity values (OAVs) compared to others [16]. Huang et al. [17] used the HS-SPME-GC-MS method to analyze the volatile compounds of *R. roxburghii* juice from five regions of Guizhou Province, and identified 67 volatile compounds, among which only 10 esters, two aldehydes, one alcohol compound, and one aromatic compound were common to samples from all regions. Sheng et al. [4] utilized headspace solid-phase micro-extraction (HS-SPME) and solvent-assisted flavor evaporation (SAFE) to extract volatile compounds from *R. roxburghii* juices sourced from four different production areas. A total of 143 volatile substances and 45 odor-active substances were identified through GC-MS and GC-O analysis, respectively. Among these, esters, alcohols, and aldehydes constituted the three primary volatile substances present in the *R. roxburghii* aroma.

Recently, research on *R. roxburghii* fruit primarily focuses on fully mature fruit stages [4,18]. However, there is a scarcity of studies regarding the variations in active components and volatile substances throughout the entire ripening process of *R. roxburghii* fruit. The harvesting of *R. roxburghii* fruit is predominantly based on color, lacking a comprehensive assessment of its volatile substances and active components across multiple parameters. In recent years, increasing attention has been directed towards the quality and volatile aroma substances formation during the development of *R. roxburghii* fruit. Hence, exploring the differences in aroma and quality among *R. roxburghii* fruit at different stages of maturity holds significant importance. This study aimed to investigate the variances in active components and changes in volatile substances in *R. roxburghii* fruit at different maturity levels and conduct a comprehensive evaluation of *R. roxburghii* fruit quality through the analysis of active components and volatile organic compounds (VOCs) in *R. roxburghii* fruit. This approach aimed to elucidate the alterations in active components and aroma compounds during the maturation of *R. roxburghii* fruit, serving as a foundation for precise processing and value-added enhancements of *R. roxburghii* fruit, and providing theoretical guidance and data support for optimal harvesting and processing of *R. roxburghii* products.

## 2. Materials and Methods

### 2.1. Plant Materials and Samples

The *R. roxburghii* samples were collected from the ‘Shili Cili Gully’ in Longli County, Qiannan Prefecture, Guizhou Province, China. All *R. roxburghii* trees studied were of the variety “Guinong No. 5”. These trees received uniform treatment in terms of fertilization and watering within the same orchard, and the soil and environmental conditions were consistent across all samples of *R. roxburghii*. Three healthy *R. roxburghii* fruit trees with consistent growth conditions were chosen for harvesting. Each tree was harvested from three different orientations: east, south, and northwest, facing the sun to ensure equal representation of sampling. The *R. roxburghii* fruits were picked at 45, 65, 75, 90, and 105 days after anthesis, representing different stages of fruit maturity and labeled as I, II, III, IV, and V, respectively.

### 2.2. Chemicals and Reagents

A WST-1 sodium dodecyl sulfate (SDS) kit was obtained from Nanjing Jiancheng Bioengineering Institute in Nanjing, China. The standards (purity ≥ 99%), such as ascorbic acid, gallic acid, rutin, and sucrose, were sourced from Aladdin Biochemical Technology Co., Ltd. in Shanghai, China. The C_7_~C_40_ n-alkane mixture was of chromatographic purity and was acquired from Shanghai Amplica Standard Technical Service Co., Ltd. in Shanghai, China. All other chemical materials and reagents used were of an analytical grade.

### 2.3. Instruments and Equipment

A 101-1AB electric blast drying box, a DK-93 electric thermostatic water bath, and a SX-12-10D box-type resistance furnace were obtained from Tianjin Test Instrument Co., Ltd. in Tianjin, China. A Cary60UV-vis spectrophotometer and an Agilent 7890-5975C gas chromatograph–mass spectrometry (GC-MS) were from Agilent Technology Co., Ltd. in the Santa Clara, CA, USA. A JY-502 electronic balance was from Shanghai Puchun Measuring Instrument Co., Ltd. in Shanghai China. An MS-H280D magnetic stirrer was from Beijing Langeco Technology Co., Ltd. in Beijing China. An OSE-VX-03 TGyrate mini vortex mixing instrument was from Beijing Tiangen Biochemical Technology Co., Ltd. in Beijing China.

### 2.4. Determination of Active Ingredients in Rosa roxburghii Fruit

After the samples were transported back to the laboratory, the roots and seeds of *R. roxburghii* were first removed. The pulp was then chopped, mixed, and immediately treated with liquid nitrogen before being placed in an ultra-low temperature refrigerator set to −80 °C for rapid freezing. Prior to testing, the fruit was crushed and subsequently ground in liquid nitrogen for further analysis.

#### 2.4.1. Measurement of Total Acid in *Rosa roxburghii* Fruit

The total acid (TA) content was measured following the method described by Liang et al. [19], with slight adjustments, using 0.1 mol/L of NaOH titration. The results were reported in terms of malic acid equivalents.

#### 2.4.2. Measurement of Total Flavonoid in *Rosa roxburghii* Fruit

The content of total flavonoids (TFs) was determined using the colorimetric method with aluminum nitrate. The results were then expressed as the rutin mass concentration, following the procedures detailed by Sammani et al. [20].

#### 2.4.3. Measurement of Vitamin C in *Rosa roxburghii* Fruit

The determination of vitamin C (Vc) was conducted using the molybdenum blue colorimetric method. Initially, 5.0 g of the homogenized *R. roxburghii* sample was precisely weighed and then diluted to 100 mL with an oxalate–EDTA solution. This concoction was subsequently filtered, and 5 mL of the resulting filtrate was transferred to a 25 mL volumetric flask. To this, 0.5 mL of metaphosphoric acid–acetic acid solution was added, followed by the addition of 1 mL of 5% sulfuric acid and 2 mL of ammonium molybdate solution. The volume was brought up to 25 mL, and the solution was then double diluted before being exposed to a water bath at 30 °C for 20 min. Post cooling naturally, the absorbance at 705 nm was recorded. The Vc content, in terms of the mass of ascorbic acid, was determined using a standard curve. This experimental procedure was repeated three times for accuracy.

#### 2.4.4. Measurement of Superoxide Dismutase in *Rosa roxburghii* Fruit

The superoxide dismutase (SOD) content was determined using a WST-1 kit, which was manufactured in Nanjing Jiancheng Bioengineering Institute, Nanjing, China. SOD activity was then measured by the water-soluble tetrazolium (WST) method.

#### 2.4.5. Measurement of Total Phenolic in *Rosa roxburghii* Fruit

The total phenolic (TP) content was determined in accordance with the methodology proposed by Dragovic-Uzelac et al. [21] and the results were expressed in terms of the gallic acid mass concentration. 

#### 2.4.6. Measurement of Soluble Tannin in *Rosa roxburghii* Fruit

The tannin content was quantified using the Folin–Ciocalteu method, with results expressed as the gallic acid mass concentration [22].

#### 2.4.7. Measurement of Soluble Sugar in *Rosa roxburghii* Fruit

The soluble sugar (SS) content was quantified using the anthrone colorimetric method, as described by Yue, et al. [23], with minor modifications.

### 2.5. Determination of Relative Contents of Volatile Organic Compounds (VOCs) in Rosa roxburghii Fruit

The relative contents of volatile organic compounds (VOCs) in *R. roxburghii* fruit were measured by the Gas Chromatography-Mass Spectrometry system (7890-5975C, Agilent Corporation, Santa Clara, CA, USA) equipped with a HP-5 MS column (60 m × 250 μm × 0.25 μm, Agilent, USA). The details of this method were as follows:

Sample pretreatment: ground and mixed *R. roxburghii* fruit (2.0 g) was placed in a 20 mL headspace vial. Boiling distilled water (4.0 mL) and phenethyl acetate internal standard solution (10.0 μL) were added, followed by sealing the vial with a screw cap equipped with a silicone headspace pad. The vial was then mixed in a vortex apparatus and each sample was measured three times.

Headspace solid phase microextraction (HS-SPME) conditions: prior to chemisorption, the 50 μm DVB/CAR/PDMS fiber (Stableflex, 24 Ga, Manual Holder, 3pk, Santa Clara, CA, USA) in the extraction head (HP-5 MS) was subjected to a 30 min pretreatment at 240 °C to ensure the absence of residual substances. Subsequently, the fiber was exposed to the headspace vial containing the pre-treated sample, and was desorbed and analyzed at 50 °C in a water bath with stirring speed of 300 rpm for 30 min. Following this, the GC injector was introduced for 3 min for desorption and analysis.

Gas chromatography (GC) condition: an HP-5 capillary column (60 m × 250 μm × 0.25 μm, Agilent, USA) was utilized with an inlet temperature set at 250 °C. The temperature ramp-up procedure consisted of an initial temperature of 40 °C for 3 min, followed by a ramp to 80 °C at a rate of 5 °C/min, further increasing to 160 °C at a rate of 10 °C/min with a 30 s hold, then progressing at 2 °C/min to 175 °C, and finally at 10 °C/min to 230 °C, maintaining for 7 min. The analysis employed a non-shunt injection mode, using high purity helium carrier gas (purity ≥ 99%) with a solvent delay time of 7 min.

Mass spectrometry (MS) conditions: an electron bombardment (EI) ion source was utilized with an electron energy of 70 eV. The ion source temperature was set at 230 °C and the quadrupole temperature at 150 °C. A full scanning mode was chosen with a mass scan range (*m*/*z*) of 45 to 500 amu. 

Qualitative and quantitative analysis: the results were initially analyzed using Chemistry Book and NIST (National Institute of Standards and Technology Standard Reference Database) database queries. Simultaneously, the Retention Index (RI) of C_7_~C_40_ normal alkanes was calculated through chromatographic scanning to further identify the VOCs in *R. roxburghii* fruit. The VOCs were quantified using phenethyl acetate as the internal standard, and the relative content of each compound was determined by comparing it with the area of the internal standard.

### 2.6. Relative Odor Activity Value of VOCs

The relative odor activity value (ROAV) was the ratio of the relative concentration of a VOC to the olfactory threshold of that substance in water. Threshold values were taken from the database [24,25]. The calculation formula was as follows:

$$ROAV=\frac{C_{i}}{{OT}_{i}}$$

where *C_i_* is the relative content of the compound, mg/kg; and *OT_i_* is the olfactory threshold of the compound in aqueous solution, mg/kg. 

### 2.7. Statistical Analysis 

All data are reported as mean value ± standard deviation (SD). Data statistics and analysis were conducted using the SPSS 20.0 software (SPSS Inc., Chicago, IL, USA). Figures were generated using Origin 2021 software (Origin Lab Corporation, Northampton, MA, USA), and a heatmap of VOCs was created using TBtools (Version 0.655). Orthogonal partial least squares discriminant analysis (OPLS-DA) was performed using the SIMCA 14.1 software (Version 14.10, Umea, Sweden) to calculate the importance projection (VIP).

## 3. Results and Discussion 

### 3.1. Changes of Active Components of R. roxburghii Fruit at Different Stages of Maturity

**Figure 1:**
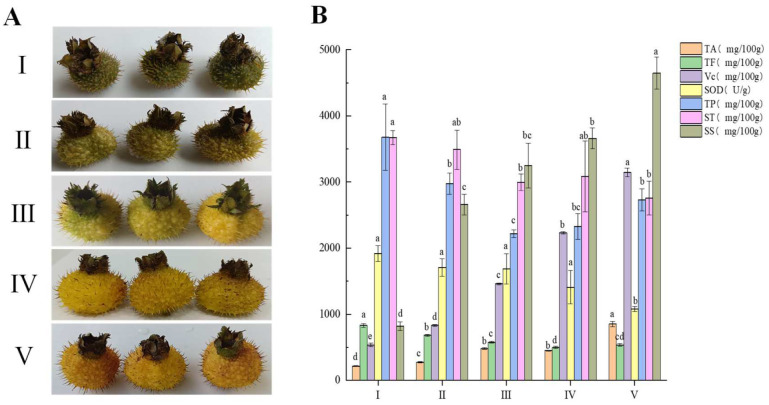
(**A**) Pictures of *R. roxburghii* fruit at different stages of maturity. The numerals I, II, III, IV, and V represent 45, 65, 75, 90 and 105 days after anthesis, respectively. (**B**) The content of active components of *R. roxburghii* fruit at different stages of maturity. TA, total acid; TF, total flavonoid; Vc, vitamin C; SOD, superoxide dismutase; TP, total phenolic; ST, soluble tannin; SS, soluble sugar. The different lowercase letters above the same color bars represent significant differences (*p* < 0.05).

Figure 1A presented images of *R. roxburghii* fruit at different stages of maturity, denoted by I, II, III, IV, and V corresponding to 45, 65, 75, 90, and 105 days after anthesis, respectively. Figure 1B depicted the variations in active compounds of *R. roxburghii* fruit across different maturity stages. During maturation, there was a significant increase (*p* < 0.05) in TA, Vc, and SS levels, while TF, SOD, and ST concentrations significantly decreased (*p* < 0.05). The levels of TP showed fluctuations with the progress of maturity.

The acidity and palatability of *R. roxburghii* fruit during the ripening process could be understood by analyzing the variation in total acidity (TA), which was the main index for evaluating the sourness of *R. roxburghii* [11]. As illustrated in Figure 1B, the TA content of *R. roxburghii* fruit significantly increased (*p* < 0.05) from stage I (214.19 mg/100 g) to stage V (850.05 mg/100 g) as it matured. Notably, there were rapid enhancements at stages III and V, with TA levels almost doubling compared to the preceding stages II and IV, respectively. Vitamin C (Vc), the main active substance in *R. roxburghii* fruit, played a crucial role in generating antioxidant properties of *R. roxburghii* [26]. The concentration of Vc in *R. roxburghii* fruit significantly increased (*p* < 0.05) from stage I (535.42 mg/100 g) to stage V (3144.72 mg/100 g) as it matured (Figure 1B), consistent with findings from Xu et al. [26]. During the maturation process of *R. roxburghii* fruit, as the cells of the fruit expanded, L-ascorbic acid (AsA), also known as Vc, was rapidly accumulated in the cytoplasm primarily through the ascorbate–glutathione cycle. Furthermore, dehydroascorbate reductase (DHAR) played a crucial role in the regeneration of AsA in the fruit and in the regulation of AsA levels within the ascorbate–glutathione cycle [27]. Vc content showed a slow increase in the initial two stages I (535.42 mg/100 g) and II (828.84 mg/100 g) due to the presence of ascorbate peroxidase (APX) and ascorbic acid oxidase (AAO), both known to be negatively correlated with Vc accumulation in plant cells [28,29]. Additionally, Yang [30] noted that these two enzymes were only detectable in the early stages of fruit ripening (around 65 days after anthesis), becoming almost undetectable after around 70 days. This explained the rapid increase in Vc content from stage III (1459.94 mg/100 g) onwards, which was also demonstrated by Huang, Xu, and Deng [27]. Soluble sugars (SSs) are the primary compounds responsible for the sweetness in fruits and are key quality indicators in assessing fruits and vegetables [31]. The SS content in *R. roxburghii* fruits significantly increased (*p* < 0.05) from stage I (819.67 mg/100 g) to stage V (4648.45 mg/100 g) as they matured. During the ripening process of *R. roxburghii* fruits, complex macromolecular carbohydrates are broken down into smaller soluble carbohydrates, leading to a gradual rise in SS content. Notably, there was a rapid increase in SS content at stage II (2658.29 mg/100 g), where it was more than three times that of stage I (819.67 mg/100 g). Subsequently, the SS content continued to steadily increase from stage II onwards, eventually reaching the highest value of 4648.45 mg/100 g at stage V.

SOD, which is a common endogenous and antioxidant enzyme in fruits, has a great impact on the texture, flavor and color of fruits [32,33]. In contrast to the accumulation of TA, Vc, and SS, the content of SOD showed a slowly significant decrease (*p* < 0.05) from stage I (1918.30 U/g) to stage V (1080.86 U/g) during the ripening process of *R. roxburghii* fruit. The ripening process of *R. roxburghii* fruit involves an oxidative phenomenon that leads to the accumulation of reactive oxygen species (ROS) such as superoxide radical, hydrogen peroxide, and hydroxyl radical [34]. However, excessive levels of ROS can cause oxidative damage to biomolecules like DNA, proteins, and lipids, ultimately resulting in cell death [35]. In order to prevent toxic concentrations of ROS, taller plants produce superoxide dismutase (SOD) to efficiently convert ROS into H_2_O_2_ and O_2_ [36]. As *R. roxburghii* fruit matures, the consumption of SOD increases to suppress the production of ROS. Soluble tannin (ST) is a crucial component contributing to the astringency of *R. roxburghii* fruit [37]. Research indicates that the higher the tannin content, the more pronounced the astringent taste [12]. Throughout the ripening stages of *R. roxburghii* fruit, there was a significant decrease (*p* < 0.05) in ST content from stage I (3674.12 mg/100 g) to stage V (2759.63 mg/100 g). This reduction could be attributed to tannin polymerization, leading to the formation of insoluble tannins as the fruit matures [38]. Previous studies have shown that tannin and soluble pectin formed during fruit ripening tended to form complexes, resulting in the production of high molecular weight insoluble tannins that bound closely to the cell wall [39,40]. Consequently, the ST content decreases progressively during the ripening process. The total flavonoid (TF) and total phenol (TP) levels are key contributors to the antioxidant properties of *R. roxburghii* fruit, with their levels being influenced by factors such as growth environmental conditions and maturity stages [2,26]. During the ripening process of *R. roxburghii* fruit, the TF content significantly decreased (*p* < 0.05) from stage I (829.65 mg/100 g) to stage V (533.37 mg/100 g). In contrast, the TP content exhibited a fluctuating pattern, initially decreasing significantly (*p* < 0.05) from stage I (3678.71 mg/100 g) to stage III, reaching its lowest value of 2220.89 mg/100 g, before gradually increasing to 2729.40 mg/100 g at stage V.

### 3.2. The Composition and Changes of VOCs in the Ripening Process of R. roxburghii Fruit

VOCs play a crucial role in assessing fruit aroma and significantly influence consumer acceptance. A total of 145 VOCs were identified in *R. roxburghii* across five different ripening stages using HS-SPME-GC-MS (Table 1). The identified compounds were categorized as follows: 19 aldehydes, 17 alcohols, 30 esters, three acids, 14 ketones, 44 alkenes, nine alkanes, four phenols, and five other compounds. Notably, the VOCs present in *R. roxburghii* exhibited differences in composition and content across the various ripening stages. Importantly, six compounds (four aldehydes, one alcohol, and one ester) were consistently present at all five ripening stages: 2-(E)-hexenal, 1-nonanal, (E)-2-pentenal, hexanal, 1-hexanol and hexyl acetate. These findings are comparable to the principal volatiles identified during the maturation of peach [41] and kiwifruit [42]. Similar substances are primarily C_6_ volatile compounds, likely due to the fact that these compounds, commonly found in fresh fruits, are produced mainly through the action of lipoxygenase on C_18_ unsaturated fatty acids, such as linoleic acid and linolenic acid [43].

**Figure 2:**
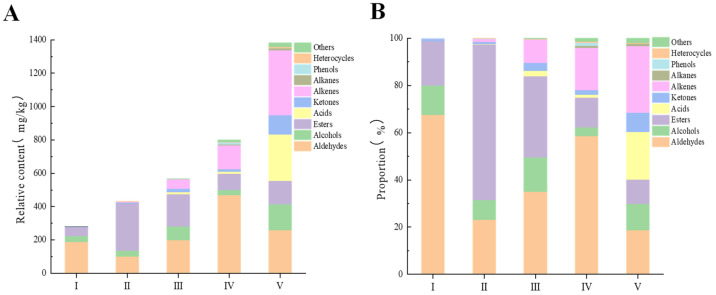
The relative contents (**A**) and percentage contents (**B**) of different types of volatile organic compounds (VOCs) in *R. roxburghii* fruit at different ripening stages. The numerals I, II, III, IV, and V represent 45, 65, 75, 90 and 105 days after anthesis, respectively.

As indicated in Figure 2A, the abundance of VOCs increased with the ripening period of *R. roxburghii* fruits. This increase might be attributed to prolonged exposure to light and the more complex metabolic processes occurring during fruit development, which ultimately resulted in the production of a greater variety of VOCs. In contrast, the data presented in Figure 2B show that aldehydes, alcohols, esters, and alkenes comprised over 90% of the total VOCs present in *R. roxburghii* fruit during the initial four stages of development. However, at stage V, these compounds accounted for approximately 68% of the total VOCs, while ketones and acids represented about 28% of the total VOCs.

#### 3.2.1. Dynamic Changes of Volatile Aldehydes during the Ripening Process of *R. roxburghii*

Linear and branched-chain aldehydes of C_6_ and C_9_, which are frequently detected in fresh fruits, are primarily produced from C_18_ unsaturated fatty acids (e.g., linoleic and linolenic acids) through three metabolic pathways: α-oxidation, β-oxidation, and lipoxygenase [43]. Aldehydes constitute one of the main aroma components of *R. roxburghii* fruits, and their accumulation varies with the ripening stage. As illustrated in Figure 2A,B, the aldehyde content peaked at stage IV (469.63 mg/kg) and was lowest at stage II (100.00 mg/kg). The percentage of aldehydes was highest at stage I (68%), whereas it was lowest at stage V (19%). During metabolism, aldehydes were converted to other substances and to each other. As *R. roxburghii* fruits continued to ripen, the aldehyde levels increased while ester levels decreased. Similar findings were reported by Li, et al. [44] and Ortiz, et al. [45]. As presented in Table 1, hexanal and 2-(E)-hexenal were the most abundant aldehydes, exhibiting a trend of first decreasing, then increasing, and subsequently decreasing again. These two C_6_ compounds are believed to be formed through the lipoxygenase pathway in fatty acid metabolism [8].

#### 3.2.2. Dynamic Changes of Volatile Alcohols during the Ripening Process of *R. roxburghii*

Alcohols in plants are derived from sugar metabolism, amino acid decarboxylation/dehydrogenation, and fatty acid oxidation, resulting in a diverse array of compounds that are significant aromatic components of fruits [46,47]. As illustrated in Figure 2A,B, the relative content of alcohols generally exhibited an increasing trend with advancing fruit maturity in *R. roxburghii*, peaking at stage V (156.11 mg/kg), which was approximately 4.5 times higher than the level observed at stage I (34.76 mg/kg). The proportion of alcohols in *R. roxburghii* fruit varied across maturity stages, with stage IV exhibiting the lowest proportion at around 4%. As *R. roxburghii* fruit matured, aldehydes decreased during stages II and V, while alcohols increased, indicating an inverse relationship between the two volatiles. As detailed in Table 1, 2-(E)-hexenal was least abundant at stages III and V, whereas (3E)-3-hexen-1-ol, detected exclusively at these two stages, was the most abundant alcohol in the fruit. This compound is produced through the metabolism of 2-(E)-hexenal by isomerase and ethanol dehydrogenase [43]. 1-Hexanol are present throughout all stages of fruit ripening and might result from the reduction in hexanal by alcohol dehydrogenase (ADH). It has been demonstrated that during fruit ripening, lipoxygenase (LOX) and hydroperoxide lyase (HPL) convert linoleic and linolenic acids into C_6_ aldehydes, which are subsequently reduced by ADH to form the corresponding C_6_ alcohols [41,48].

#### 3.2.3. Dynamic Changes of Volatile Esters during the Ripening Process of *R. roxburghii*

Esters in fruits are generally derived from straight or branched-chain carboxylic acid esters originating from fatty acid and amino acid pathways [43]. As illustrated in Figure 2, there were notable differences in the accumulation of esters in *R. roxburghii* at various ripeness levels. The ester content at stage I was significantly lower than that in the other four stages. The highest ester content was observed at stage II, measuring approximately 283.97 mg/kg, which was about five times greater than the content at stage I (52.69 mg/kg). Additionally, the proportion of esters at stage II was the largest, accounting for around 66%, whereas the proportion at stage V was the lowest, at approximately 10%. The accumulation of esters in the *R. roxburghii* fruit ripening process contrasted with the accumulation of aldehydes, and this finding aligned with the conclusions of Menager, I et al. [49], who noted similar variations during the ripening of strawberries. In the fatty acid pathway, unsaturated fatty acids, such as linoleic and linolenic acids, can be converted by lipoxygenase (LOX) and hydroperoxide lyase (HPL) to form aldehydes, which are subsequently reduced by alcohol dehydrogenase (ADH) to yield the corresponding alcohols. These alcohols then undergo acylation to form esters, a process catalyzed by alcohol acyltransferase (AAT) [50,51]. As shown in Table 1, hexyl acetate was detected at all stages, likely resulting from the esterification of acetic acid with hexanal. (Z)-3-hexenyl acetate was the most abundant ester, derived from (3E)-3-hexen-1-ol, which involves a series of reactions, including isomerization, acyltransferase activity, and the involvement of acetyl coenzyme A [8,52]. This indicates that hexyl acetate and (Z)-3-hexenyl acetate are the primary components contributing to the aroma of *R. roxburghii* esters.

#### 3.2.4. Dynamic Changes of Volatile Acids during the Ripening Process of *R. roxburghii*

The primary source of volatile acids in fruits is the oxidation of fatty acids; furthermore, the interaction between the acid fraction and acyl carrier proteins during fatty acid synthesis can also produce volatile acids [43,47]. As illustrated in Figure 2, the concentration of acids in *R. roxburghii* fruit was below the detection limit at stage I and subsequently increased with maturation, ultimately reaching a maximum value of 278.19 mg/kg at stage V. This peak represented approximately 20% of the total acids, with octanoic acid being the predominant component.

#### 3.2.5. Dynamic Changes of Volatile Ketones during the Ripening Process of *R. roxburghii*

The primary pathways for the production of straight and branched chain ketones include the oxidation of alcohols, the reduction in acids, and processes associated with sugar metabolism [53]. The data presented in Figure 2 indicate that the content and percentage of ketones in *R. roxburghii* remained low across various stages of ripeness, although an overall increasing trend was observed, culminating in a maximum value of 114.73 mg/kg at stage V, where the percentage reached approximately 8%. Conversely, carbohydrates served as significant precursors for furanones. The GC-MS technique identified 4-methoxy-2,5-dimethyl-3 (2H)-furanone as the predominant ketone across different ripening stages of *R. roxburghii*, with its content consistently increasing as the fruit ripened, achieving the highest concentration of furanone compounds at stage V, measured at 50.37 mg/kg. It is noted that D-fructose-1,6-bisphosphonic acid first generates the intermediate 4-hydroxy-5-methyl-2-methylene-3 (2H)-furanone, which is subsequently converted to methoxyfuranone through the action of O-methyltransferase [54,55,56]. This finding significantly enhanced our understanding of the mechanism underlying furanone production in *R. roxburghii*.

#### 3.2.6. Dynamic Changes of Volatile Alkenes during the Ripening Process of *R. roxburghii*

Alkenes, particularly those of the C_10_ and C_15_ varieties, exert a pronounced influence on fruit flavor and floral attributes, and are predominantly synthesized from carbohydrates in the plastid and cytoplasm through enzymatic reactions involving acetyl coenzyme A and pyruvate [43,47]. Figure 2 illustrates that the alkane content and percentage exhibited a gradual increase throughout the fruit ripening process in *R. roxburghii*. At stage V, the alkene content reached a maximum value of 389.34 mg/kg, which was approximately 700 times the content observed in stage I and represented about 28% of the total. This indicated a significant increasing trend, suggesting that as the fruit matured, there was not only an increase in the quantity of alkenes but also a diversification of these compounds.

The contents of alkanes, phenolics, heterocyclic compounds, and other substances displayed variability at different ripening stages, with an overall increasing trend observed throughout the fruit ripening process. Among these compounds, heterocyclic compounds were predominantly represented by α-agarofuran, which exhibited a continuous increase in concentration as the ripening stage progressed.

### 3.3. Analysis of ROAV Values of VOCs during the Ripening Process of R. roxburghii

The aroma characteristics of 145 volatiles detected by HS-SPME-GC-MS at five ripening stages of *R. roxburghii* fruit were not determined solely by their relative content, but rather by the relative odor activity value (ROAV), which combines both the content and odor threshold.The ROAV is a widely used method for evaluating the contribution of volatile substances to the overall aroma. According to Zhu et al. [57], substances with an ROAV of >1 are perceived by humans and contribute to the overall aroma. In total, 53 VOCs with an ROAV of >1 were identified (Table 2). These substances were categorized into 17 aroma categories, including flower, fruit, grass, green, fat, soap, wood, caramel, balsamic, sweet, mint, mushroom, citrus, metal, pungent, lemon, and sweat aromas. This finding highlightes the diversity and complexity of the aroma profiles of *R. roxburghii* fruit during ripening.

**Figure 3:**
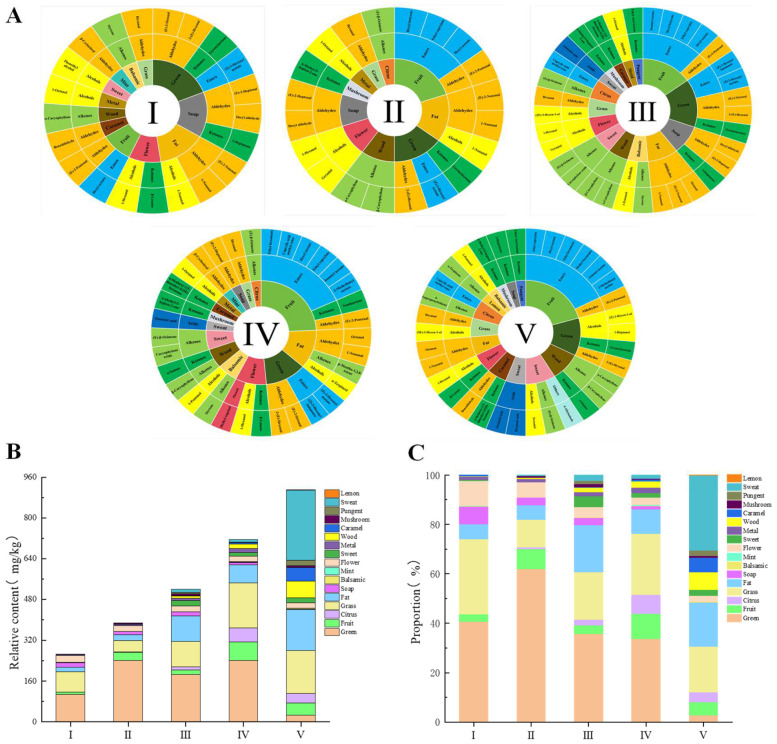
Aroma descriptions and the relative contents of VOCs with an ROAV of >1 in *R. roxburghii* fruit at different ripening stages. (**A**) Characteristic flavor wheels; (**B**) relative contents; and (**C**) percentage contents.

As illustrated in Figure 3A, the odors associated with fruit, green, grass, flower, and so on underwent significant changes during the ripening of *R. roxburghii*. At stage I, a total of 12 aroma types were identified, predominantly characterized by floral and fruity notes, with a unique mint flavor (β-cyclocitral contribution, ROAV > 100) that was exclusively observed at stages I and IV. At stage II, the number of aroma categories decreased to 10, with the introduction of citrus and mushroom flavors, which were derived from (Z)-β- ocimene and 6-methyl-5-hepten-2-one, respectively. Stage III saw an increase to 15 aroma categories, incorporating pungent and sweat flavors from ethyl vinyl ketone and octanoic acid, respectively; notably, pungent flavors were present only at stages III and V. Stage IV closely resembled stage III but lacked the pungent notes, while the concentration of mint increased. At stage V, the total number of aroma types rose to 17, although mint and metal flavors were absent, featuring a distinctive lemon flavor contributed by α-terpinene. Figure 3B,C further illustrates that during the ripening of *R. roxburghii* fruits, both the categories of aroma and their relative concentrations increased. The primary characteristic aromas, which included green, fruit, grass, fat, flower, soap, and wood, accounted for over 85% of the aroma profiles at the first four stages and nearly half at stage V.

Combined with Figure 3B,C, and Table 2, it was evident that the green aroma primarily consisted of aldehydes, alcohols, esters, and ketones, with 2-(E)-hexenal and (Z)-3-hexenyl acetate being the most significant contributors (ROAV > 100); notably, (Z)-3-hexenyl acetate was undetectable at stage V. Alcohols contributing to green aroma volatiles were exclusively present at stage V, specifically 1-heptanol and (Z)-2-hexen-1-ol. The fruit aroma was derived from seven esters alongside a few aldehydes and ketones, with evidence indicating that esters predominantly contribute to the fruit aroma in the later stages of fruit development [43]. Isoamyl acetate and ethyl hexanoate emerged as the primary contributors to the later stages of *R. roxburghii* ripening, both exhibiting an ROAV of >1000, while methyl hexanoate contributed to the fruit flavor only at stage V, with an ROAV of >100. The grass flavor was mainly attributed to aldehydes (hexanal) and alcohols ((3E)-3-Hexen-1-ol), with hexanal being the principal contributor to the grass flavor, present at all stages and having an ROAV of >1000. Fat aromas were predominantly formed by aldehydes, followed by alcohols and alkenes, with 1-nonanal being the major contributor (ROAV > 10,000). (E)-2-nonenal and octanal dominated the early and late phases, respectively, each with an ROAV of >1000. α-terpineol and p-mentha-1,3,8-triene were only detected at stage IV. The flower aroma was primarily provided by alcohols, ketones, and phenols, with 1-hexanol (ROAV > 1000) and β-Lonone (ROAV > 10,000) as the main volatiles, while methyl eugenol was unique to stage IV. The aroma of soap was primarily attributed to aldehydes (decyl aldehyde, (E)-2-heptenal) and ketones (2-heptenone). Decyl aldehyde and (E)-2-heptenal were concentrated during the prophase, with an ROAV greater than 100. Overall, the concentration of aldehydes contributing to the green and soap aromas decreased as fruit ripening progressed. Research indicates that aldehydes are predominantly concentrated in the early stages of fruit ripening and diminish as ripening continues [43]. In contrast, the aroma of wood was mainly derived from alkenes (α-caryophyllene, β-caryophyllene) and ketones (α-ionone). The overall aroma of wood increased, along with the content of VOCs responsible for this wood aroma (Figure 3B). Notably, β-caryophyllene was absent during the initial stage, but its concentration peaked with the advancement of maturation, achieving an ROAV greater than 100 at stage V.

There was a distinct pattern in the types of aromas specific to various classes, such as citrus, caramel, and sweet, which were concentrated at the late stage. The citrus aroma was primarily contributed by alkenes and esters, while the caramel aroma was predominantly characterized by 4-methoxy-2,5-dimethyl-3 (2H)-furanone. The sweet aroma was derived from a variety of substances, with 4-allylanisole being particularly notable at stage V, where it exhibited an ROAV greater than 1000.

### 3.4. Differential Volatile Aroma Screening and OPLS-DA Analysis of VOCs during Ripening of R. roxburghii

**Figure 4:**
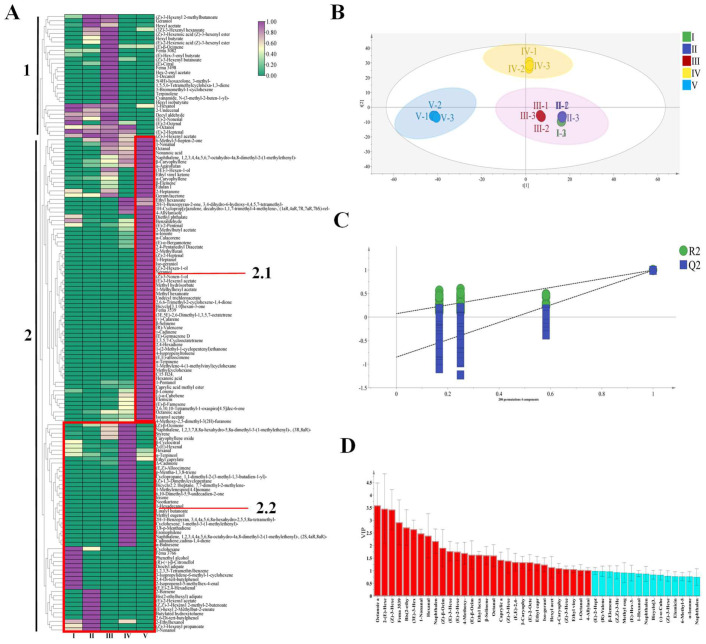
Clustering heat map and OPLS-DA analysis of VOCs in *R. roxburghii* fruit at different ripening stages. (**A**) Clustering heat map; (**B**) score plot (R^2^X = 0.995, R^2^Y = 0.921, Q^2^ = 0.817); (**C**) 200 permutation test cross-validation plot (R^2^ = 0.0722, Q^2^ = −0.85); and (**D**) VIP diagram, red bars represent VOCs with VIP value over 1.

The OPLS-DA analysis of 145 volatile substances in *R. roxburghii* fruit revealed that the first three stages (I, II, and III) were located in the same quadrant, indicating a similarity in the volatile components of the fruits. In contrast, stages IV and V were distributed across different quadrants, resulting in the formation of three distinct groups (Figure 4B): the first three stages (I, II, and III), stage IV, and stage V. Additionally, Figure 4A illustrates the significant changes in *R. roxburghii* across five maturation stages through the quantitative visualization of VOCs, thereby confirming the aforementioned classification. For additional information regarding the VOCs depicted in Figure 4A, please refer to Figure S1 in the Supplementary File. The 145 VOCs of *R. roxburghii* exhibited marked differences at various stages of ripening. Green and purple colors indicate the decrease and increase in substance content, respectively, directly reflecting the changes occurring during the ripening process of *R. roxburghii*. Through the cluster heat map, the 145 VOCs are organized into two distinct clusters, labeled 1 and 2, with cluster 2 further subdivided into two subclusters: 2.1 and 2.2.

Cluster 1 was characterized by the changes in VOCs during the initial three stages. In this phase, the concentrations of aldehydes and alcohols gradually decreased, while the levels of esters significantly increased. Research indicates that lipid metabolism serves as a crucial source of aldehydes and alcohols, which not only contribute directly to fruit aroma but also act as precursors for the synthesis of volatile esters [58]. Therefore, compounds with similar biosynthetic pathways are often clustered together. In cluster 1, we identified 12 hexyl esters and their corresponding prohexanols, which are derived from the metabolism of oleic and linoleic acids [59,60]. Figure 4A illustrates that the content and variety of ester substances continued to rise during the early ripening stage of *R. roxburghii* fruit. As shown in Table 2, the first three stages predominantly featured flower (1-hexanol, geraniol), fruit (hexyl butyrate, hexyl acetate), green ((E)-2-octenal, (Z)-3-hexenyl acetate, and (Z)-3-hexenyl butanoate), and fat ((E)-2-heptenal, decyl aldehyde) compounds.

Cluster 1 was notable for the formation of small clusters at stage III, which comprised 14 distinct volatiles. Among these, seven substances were unique to this stage: cyanamide, N-(3-methyl-2-buten-1-yl)-, hexyl isobutyrate, fema 3498, and so on. In conjunction with the findings presented in Table 2, the predominant aroma characteristic of this stage was a sweet aroma, primarily dominated by (E)-β-ocimene (ROAV > 100), alongside a notable green aroma, primarily derived from (Z)-3-hexenyl butanoate (ROAV > 1). Collectively, these components contributed to the distinctive aroma profiles of *R. roxburghii* fruit at stage III.

Figure 4A illustrates that cluster 2.2 was further subdivided into three sub-clusters, which corresponded to the I, II, and IV stages of *R. roxburghii* ripening, respectively. During stage I, nine volatiles (including 2-Isopropenyl-5-methylhex-4-enal, phenethyl alcohol, and (E,E)-2,4-hexadienal, etc.) were significantly clustered and exhibited higher concentrations than in the other stages. Notably, (E,E)-2,4-hexadienal and phenethyl alcohol were particularly prominent, with phenethyl alcohol having an ROAV greater than 1, contributing to the fruit’s sweet and aromatic flavor. As the fruit progresses into stage II, esters became the dominant volatiles, consistent with previous analyses. Seven volatiles (including bis (2-ethylhexyl) adipate, (E)-2-hexenyl acetate, and 2,6-di-tert-butylphenol, etc.) that were unique to this stage could serve as distinguishing markers to differentiate stage II from the other stages. In particular, 1-nonanol (ROAV > 100) imparted a significant fat flavor during this stage.

The volatiles identified in stage IV were predominantly alkenes, with (Z)-β- ocimene being particularly prominent, exhibiting an ROAV greater than 1000, which contributed to the fruit’s strong citrus aroma. Notable compounds such as styrene (ROAV > 100), caryophyllene oxide (ROAV > 1), and p-mentha-1,3,8-triene (ROAV > 1) were also present, imparting balsamic, sweet and fat notes, respectively. Although the presence of aldehydes was limited, β-cyclocitral, 2- (E)-hexenal, and hexanal were found at high levels, each exhibiting distinctive characteristics: both 2- (E)-hexenal and hexanal were notably high, with ROAVs exceeding 1000, contributing fruit and grass flavors, respectively. Interestingly, the concentrations of these aldehydes demonstrated a pattern of initial increase followed by a decrease during the ripening process of *R. roxburghii*, which might be associated with the catalytic effect of 2-enal reductase and the interaction of aldehydes with amines and sulfhydryl groups in proteins [61,62]. Regarding esters, while their presence was limited at this stage, ethyl caprylate significantly enhanced the fruit flavor (ROAV > 1000). The synergistic effect of these volatiles collectively contributed to the unique flavor profile of *R. roxburghii* fruits at stage IV.

In the cluster analysis of cluster 2.1, stage V exhibited a distinctive composition of volatile substances, predominantly characterized by alkenes, esters, and ketones. Among the alkenes, terpenes are the principal secondary products of the acetyl-CoA metabolic pathway in plants [63,64]. These terpenes contributed complex aromas, including wood, citrus, and lemon, and were represented by compounds such as β-caryophyllene, α-caryophyllene, 4-isopropenyltoluene, and α-terpinene. Esters primarily enhanced fruit and citrus aromas, with ethyl hexanoate and isoamyl acetate exhibiting ROAV values significantly exceeding 1000, thereby serving as the main flavor contributors at this stage. Additionally, 2-methylbutyl acetate, methyl hexanoate and caprylic acid methyl ester were present at significant levels (ROAV > 100), further enriching the aroma profile of *R. roxburghii* fruit. Ketones were closely correlated and categorized together, predominantly comprising fatty acid derivatives. Compounds such as 6-methyl-5-hepten-2-one, ethyl vinyl ketone, and 2-heptanone exhibited a range of aromas, including mushroom, pungent, and soap, respectively. High ROAV components such as α-ionone, β-lonone, and 4-methoxy-2,5-dimethyl-3 (2H)-furanone impart wood, flower, and caramel aromas, respectively, and are generally considered direct products of the oxidative cleavage of carotenoids [65]. Furthermore, sweet aromas were primarily attributed to neraniol, benzaldehyde, 4-methoxy-2, 5-dimethyl-3 (2H) -furanone, and 4-allylanisole, with 4-allyl anisole being particularly noteworthy due to its low threshold and high ROAV value (>1000), making it a significant sweetening component. However, stage V was also associated with the emergence of undesirable odors, as the levels of acids such as nonanoic acid, octanoic acid and hexanoic acid peaked during this stage, which negatively impacted the overall flavor.

As a by-product of fatty acid metabolism, alkanes are primarily derived from fatty acids through the fatty acyl-CoA pathway, which is characterized by a low boiling point and minimal contribution to aroma [66]. Consequently, this pathway does not significantly influence the aroma characteristics of *R. roxburghii* fruit. In this study, we observed that the majority of hydrocarbons, particularly long-chain alkanes, were predominantly present in the IV and V maturity stages. The specific cause underlying this phenomenon warrants further investigation.

Through cluster heat map analysis, the fruit of *R. roxburghii* exhibited a distinctive distribution of VOCs across five different ripening stages, which could serve as a landmark chemical fingerprint for each stage. It is worth noting that at stages IV and V, a large amount of alkene accumulation acted as an effective biomarker, distinguishing these two stages from the previous stages. In contrast, the content of volatile esters exhibited a sharp increase at stages II and III, with these changes closely correlating with the enhancement of fruit, flower, and pleasant aromas. This correlation further corroborated the consistency of the data presented in Table 1, with the aroma profiles depicted in Figure 2 and Figure 3. It was important to highlight that the clustering pattern revealed through the abundance model of volatile substances aligned with the results of the OPLS-DA analysis (Figure 4B), thereby enhancing our understanding of the changes in volatile substances during the ripening process of *R. roxburghii* fruit. However, further in-depth exploration and verification are required to determine whether the specific volatile substances at each stage are adequate as characteristic markers for that stage.

To elucidate the trend of VOCs during the ripening of *R. roxburghii* and to identify the VOCs responsible for changes in aroma, OPLS-DA was employed (Figure 4B). Concurrently, 200 permutation tests were conducted to assess the model’s accuracy. The results indicated that R^2^X was 0.995, R^2^Y was 0.998, and Q^2^ was 0.996. Both R^2^ and Q^2^ values exceeding 0.5 suggest that the model fitting results were acceptable, demonstrating that the established model possessed strong interpretive and predictive capabilities [67]. Consequently, the model effectively elucidated the relationship of VOCs in *R. roxburghii* fruit across different ripening stages. Following the 200 permutation tests, as illustrated in Figure 4C, the intersection of the blue regression line with the vertical axis of Q^2^ was below zero, indicating that the model was not overfitted and that the validation was robust; thus, the results were deemed suitable for the identification and analysis of various ripening stages of *R. roxburghii*. The OPLS-DA diagram clearly differentiated the distinct ripening stages of *R. roxburghii* fruit, particularly stages IV and V. Stages I, II, and III were relatively close to one another, all situated in the fourth quadrant, suggesting that their volatile compounds were similar. Furthermore, Figure 2 revealed that stage IV was characterized by a richness in aldehydes, alkenes, and phenols, while stage V represented the most mature stage, abundant in alcohols, acids, ketones, alkenes, and alkanes, thereby making these two stages highly distinguishable in the figure. In contrast, stages I, II, and III were predominantly composed of aldehydes, alcohols, and esters.

In addition, the VIP was employed to identify the primary volatile organic compounds responsible for aroma changes during the ripening process of *R. roxburghii*. A VIP value greater than 1 indicated that the corresponding variable was a key component of the discriminant model; furthermore, a larger VIP value signified a more significant difference between the variables at various maturity stages [68]. Utilizing the screening criteria of *p* < 0.05 and VIP > 1, we identified 30 key differentially volatile aroma components across different ripening stages of *R. roxburghii* (Figure 4D). For more details of VOCs with VIP > 1, please refer to Figure S2 in the Supplementary File. These components comprised six types of aldehydes (such as 2- (E)-hexenal, (E,E)-2,4-hexadienal, 1-nonanal, etc.), three alcohols (1-octanol, iso-geraniol, and (3E)-3-hexen-1-ol), 10 esters (such as (E)-3-hexenyl acetate, hexyl acetate, (E)-2-hexenyl acetate, etc.), one acid (octanoic acid), two types of ketones (4-methoxy-2,5-dimethyl-3 (2H)-furanone and ethyl vinyl ketone), and seven alkenes ((Z)-β-ocimene, β-caryophyllene, α-caryophyllene, (E)-β-ocimene, etc.), along with one other compound (4-allylanisole). Notably, 2-(E)-hexenal, 1-nonanal, and hexanal, which were the principal aldehyde components, persisted and significantly contributed throughout the maturation process. 1-Octanol served as the primary volatile aroma component among the alcohols. Hexyl acetate, (Z)-3-hexenyl acetate, and ethyl caprylate were the most critical substances among the esters, with hexyl acetate and (Z)-3-hexenyl acetate being particularly prominent. Octanoic acid was identified as the main component of acids, while β-caryophyllene and α-caryophyllene were recognized as key volatile components of olefins. In summary, these differential compounds were predominantly ester compounds, which played a crucial role in the aroma formation of *R. roxburghii* fruit.

Combined with the OPLS-DA results, a total of 21 differentially volatile aromas were identified that met the criteria of VIP > 1 and ROAV > 1. These included 2- (E)-hexenal, (Z)-3-hexenyl acetate, (E)-2-octenal, (Z)-3-hexenyl butanoate, hexyl acetate, ethyl caprylate, ethyl Hexanoate, (Z)-β-ocimene, caprylic acid methyl ester, hexanal, (3E)-3-hexen-1-ol, octanal, 1-nonanal, (E)-β-ocimene, 4-allylanisole, 1-octanol, α-Caryophyllene, β-Caryophyllene, 4-methoxy-2, 5-dimethyl-3 (2H)-furanone, ethyl vinyl ketone, and octanoic acid. These compounds were considered as the key volatile constituents influencing the aroma of *R. roxburghii* fruit at the ripening stage and played a significant role in assessing the aroma characteristics across different ripening phases.

## 4. Conclusions

In this study, we analyzed the trends of seven key active components during the ripening process of *R. roxburghii* fruits. Our findings indicated that total acids (TAs), vitamin C (Vc), and soluble sugars (SSs) increased significantly (*p* < 0.05), while total flavonoids (TFs), superoxide dismutase (SOD), and soluble tannins (STs) decreased significantly (*p* < 0.05). Total phenol (TP) levels exhibited fluctuations, first decreasing and then increasing throughout the ripening process. A total of 145 VOCs were identified using HS-SPME-GC-MS technology, mainly comprising aldehydes, alcohols, esters, and alkenes. As *R. roxburghii* matured, both the variety and overall amount of VOCs in the fruit increased, with a significant escalation in the levels of acids, ketones, and alkenes. Through the calculation of ROAV values for these VOCs, 53 principal substances were recognized, which featured aromas including fruit, citrus, green, caramel, grass, flower, sweet, soap, wood, and fat notes. The scents of citrus, caramel, sweet, and wood were largely concentrated in the later developmental phases of *R. roxburghii* fruit ripening. Analysis via a cluster heatmap showed distinct patterns of VOC distribution throughout five maturity stages, functioning as unique chemical signatures for each phase. Notably, stages IV and V were primarily defined by an abundance of alkenes. OPLS-DA analysis divided the ripening progression of *R. roxburghii* fruit into three sections: the first section included the initial three stages (I, II, and III), the second section related to the fourth stage (IV), while the third section involved the fifth stage (V). Following the VIP > 1 criterion, a total of 30 significant differential VOCs were identified across the five stages, predominantly consisting of ester compounds, which notably affected the aroma profiles of *R. roxburghii* fruit. By combining the VIP > 1 and ROAV > 1 criteria, 21 differential VOCs were further recognized as vital contributors to the aromatic transformations occurring in *R. roxburghii* fruits during their ripening. However, the molecular processes responsible for aroma metabolism in *R. roxburghii* remain poorly understood, highlighting the need for further studies to clarify these metabolic pathways. Future studies should utilize gas chromatography–olfactometry (GC-O) to identify the characteristic aromas present in *R. roxburghii* fruit. Additionally, multi-omics approaches should be integrated to further investigate the mechanisms underlying the formation of key aromas in *R. roxburghii* fruit throughout the maturation process.

## Figures and Tables

**Table 1:** HS-SPME-GC-MS analysis results of VOCs in *R. roxburghii* fruit at different ripening stages.

No.	Compounds	RT	RI	LRI	Relative Content (mg/kg)				
					I	II	III	IV	V
1	(E)-2-Pentenal	9.582	744	ND	4.34	2.20	2.80	4.02	8.32
2	Hexanal	10.962	801	806.1	81.13	43.26	49.14	177.11	66.54
3	2- (E)-Hexenal	12.733	847	861.7	55.46	21.12	19.45	209.15	18.37
4	(E,E)-2,4-Hexadienal	14.399	931	921.4	4.58	ND	ND	ND	ND
5	(Z)-2-Heptenal	15.389	ND	960.5	ND	ND	ND	ND	1.95
7	(E)-2-Heptenal	15.57	978	967.6	16.54	9.86	11.28	9.46	ND
6	Benzaldehyde	15.751	961	968.4	1.48	0.09	ND	0.60	2.26
8	Octanal	16.494	1002	1005.0	ND	ND	20.81	9.97	57.75
9	Cyanamide, N- (3-methyl-2-buten-1-yl)-	17.76	ND	1066.3	ND	ND	4.82	ND	ND
10	(E)-2-Octenal	17.846	1064	1070.5	5.59	ND	4.72	0.93	ND
11	2-Isopropenyl-5-methylhex-4-enal	18.255	ND	1090.3	0.33	ND	ND	ND	ND
12	1-Nonanal	18.598	1105	1115.6	13.18	19.31	77.19	56.64	102.94
13	Fema 3766	19.712	1162	1168.7	0.50	ND	ND	ND	ND
14	(E)-2-Nonenal	19.816	1164	1174.4	0.74	0.39	0.91	ND	ND
15	Decyl aldehyde	20.645	1204	1218.8	2.33	2.26	4.04	ND	ND
16	β-Cyclocitral	21.178	1222	1244.7	0.66	ND	ND	1.74	ND
17	(E)-Citral	21.911	1269	1285.0	ND	0.12	0.57	ND	ND
18	2-Undecenal	23.853	1359	1378.2	1.90	1.10	1.63	ND	ND
19	Fema 3082	23.91	ND	1378.2	0.27	0.30	1.11	ND	ND
Aldehydes (19)					189.01	100.00	198.47	469.63	258.13
20	1-Pentanol	9.648	763	ND	ND	ND	0.36	0.21	3.20
21	(3E)-3-Hexen-1-ol	12.571	852	864.1	ND	ND	50.71	ND	102.41
22	(Z)-2-Hexen-1-ol	12.857	865	867.6	ND	ND	ND	ND	0.84
23	1-Hexanol	12.914	867	869.4	26.36	23.38	21.98	7.97	14.41
24	1-Heptanol	15.665	968	971.4	ND	ND	ND	ND	2.11
25	2-Ethylhexanol	17.179	1031	1040.0	1.75	3.14	ND	1.34	ND
26	1-Octanol	18.036	1064	1079.7	3.33	5.61	8.91	15.68	ND
27	Phenethyl alcohol	19.064	1110	1133.3	1.03	ND	ND	ND	ND
28	(Z)-3-Nonen-1-ol	19.502	1126	1157.2	ND	ND	ND	ND	0.75
29	1-Nonanol	19.969	1171	1182.2	1.31	2.86	ND	ND	ND
30	α-Terpineol	20.349	1192	1211.9	0.81	0.38	ND	2.99	0.72
31	Neraniol	20.921	1232	1233.3	ND	ND	ND	ND	1.30
32	(R)- (+)-β-Citronellol	21.054	ND	1240.2	0.17	ND	ND	ND	ND
33	Iso-geraniol	21.187	1273	1247.2	ND	ND	ND	ND	30.38
34	Geraniol	21.587	1260	1265.6	ND	0.46	0.40	ND	ND
35	1-Decanol	21.835	1279	1281.0	ND	ND	0.30	ND	ND
36	1-Hexadecanol	22.406	1876	1309.5	ND	ND	ND	1.36	ND
Alcohols (17)					34.76	35.82	82.65	29.54	156.11
37	Isoamyl acetate	13.181	876	878.1	ND	ND	0.46	0.72	5.03
38	2-Methylbutyl acetate	13.257	879	880.5	ND	ND	ND	0.66	3.70
39	Methyl hexanoate	14.523	923.2	926.3	ND	ND	ND	ND	8.77
40	Methyl hydrosorbate	14.732	ND	934.5	ND	ND	ND	ND	0.38
41	Ethyl hexanoate	16.37	1000	999.2	ND	ND	ND	32.04	15.35
42	(E)-3-Hexenyl acetate	16.551	1004	1007.8	ND	ND	ND	ND	56.61
43	(Z)-3-Hexenyl acetate	16.694	1007	1016.1	46.07	218.88	142.66	29.54	ND
44	Hexyl acetate	16.818	1011	1020.7	3.45	28.04	13.71	2.82	3.21
45	Hex-2-enyl acetate	16.875	ND	1023.5	ND	ND	2.20	ND	ND
46	(E)-2-Hexenyl acetate	16.912	1017	1025.3	ND	7.47	ND	ND	ND
47	1-Methylhexyl acetate	17.284	1043	1043.3	ND	ND	ND	ND	1.35
48	(Z)-3-Hexenyl propanoate	18.626	1100	1109.8	0.88	2.07	ND	ND	ND
49	Caprylic acid methyl ester	18.912	1126	1124.9	ND	ND	0.46	0.60	35.41
50	(E)-Hex-3-enyl butyrate	20.207	ND	1195.8	0.75	ND	6.91	ND	ND
51	(Z)-3-Hexenyl butanoate	20.207	1187	1195.8	ND	ND	15.54	1.26	ND
52	Hexyl isobutyrate	20.292	1150	1200.4	ND	ND	0.40	ND	ND
53	Hexyl butyrate	20.321	1191	1201.9	ND	0.51	1.23	ND	ND
54	Ethyl caprylate	20.378	1195	1204.9	ND	0.39	0.41	31.58	4.32
55	Linalyl butanoate	21.064	1422	1240.8	ND	ND	ND	0.73	ND
56	(Z)-3-Hexenyl 2-methylbutanoate	21.083	1233.5	1241.7	0.27	1.37	1.30	ND	ND
57	Fema 3498	22.063	1240	1292.9	ND	ND	0.92	ND	ND
58	Undecyl trichloroacetate	22.244	ND	1302.1	ND	ND	ND	ND	3.81
59	(Z,Z)-3-Hexenyl 2-methyl-2-butenoate	22.996	1282.2	1336.5	ND	2.86	ND	ND	ND
60	(E)-hexyl 2-Methylbut-2-enoate	23.091	1331	1340.8	ND	0.39	ND	ND	ND
61	(3Z)-3-Hexenyl hexanoate	23.996	1380	1389.5	ND	1.01	1.51	ND	0.45
62	(Z)-3-Hexenoic acid (Z)-3-hexenyl ester	24.329	ND	1397.4	ND	2.79	5.84	ND	ND
63	(E)-2-Hexenoic acid (Z)-3-hexenyl ester	25.5	ND	1440.8	ND	0.35	0.80	ND	ND
64	Diethyl phthalate	29.622	1602.3	1606.5	1.08	ND	ND	ND	2.36
65	Bis (2-ethylhexyl) adipate	31.498	ND	1698.5	ND	17.86	ND	ND	ND
66	Dioctyl adipate	31.517	ND	1699.5	0.18	ND	ND	ND	ND
Esters (30)					52.69	283.97	194.35	99.94	140.75
67	Hexanoic acid	15.999	981	984.6	ND	ND	ND	ND	7.93
68	Octanoic acid	19.94	1178	1178.1	ND	0.96	12.23	9.85	268.93
69	Nonanoic acid	21.454	1280	1269.6	ND	ND	1.00	0.82	1.33
Acids (3)					ND	0.96	13.22	10.67	278.19
70	Ethyl vinyl ketone	7.392	680	ND	ND	ND	6.46	ND	20.51
71	2-Heptanone	13.599	893	891.6	0.37	ND	0.45	ND	0.71
72	Bicyclo [3.1.0]hexan-3-one	14.209	ND	913.9	ND	ND	ND	ND	13.48
73	5 (4H)-Isoxazolone, 3-methyl-	16.17	ND	991.3	ND	ND	0.81	ND	ND
74	6-Methyl-5-hepten-2-one	16.256	987	994.7	ND	2.89	6.51	2.71	7.09
75	4-Methoxy-2,5-dimethyl-3 (2H)-furanone	17.893	1065	1072.3	ND	ND	1.66	4.96	50.37
76	2,6,6-Trimethyl-2-cyclohexene-1,4-dione	19.426	1152	1153.1	ND	ND	ND	ND	0.42
77	Irisone	23.025	ND	1337.8	ND	ND	ND	1.92	ND
78	α-Ionone	25.633	1429	1441.9	ND	ND	ND	0.50	9.27
79	Geranylacetone	25.862	1453	1456.9	1.20	1.30	3.24	ND	4.14
80	6,10-Dimethyl-5,9-undecadien-2-one	26.014	1456	1462.8	ND	ND	ND	1.83	ND
81	β-Lonone	27.214	1488	1503.3	0.60	ND	ND	1.28	7.67
82	2H-1-Benzopyran-2-one, 3,4-dihydro-6-hydroxy-4,4,5,7-tetramethyl-	29.013	ND	1575.7	ND	ND	ND	1.64	1.07
83	Nootkartone	33.516	1800	1814.5	ND	ND	ND	0.88	ND
Ketones (14)					2.17	4.19	19.13	15.73	114.73
84	1,3,5,7-Cyclooctatetraene	13.695	ND	894.7	ND	ND	ND	ND	0.49
85	Styrene	13.885	894	900.0	0.05	ND	0.52	0.96	ND
86	2,4-Hexadiene	14.428	ND	922.5	ND	ND	ND	ND	0.56
87	1- (2-Methyl-1-cyclopentenyl)ethanone	16.855	ND	1022.5	ND	ND	ND	ND	1.40
88	3-Bromomethyl-1-cyclohexene	17.008	ND	1029.9	ND	ND	0.57	ND	ND
89	(E)-β-Ocimene	17.389	1051	1048.4	ND	ND	21.88	2.03	2.51
90	Fema 3539	17.484	1050	1053.0	ND	ND	ND	ND	99.74
91	(Z)-β-Ocimene	17.627	1040	1059.9	ND	2.90	11.09	53.77	ND
92	4-Isopropenyltoluene	18.417	1090	1098.1	ND	ND	ND	ND	0.60
93	(E,E)-alloocimene	19.055	ND	1132.8	ND	ND	ND	ND	4.26
94	(3E,5E)-2,6-Dimethyl-1,3,5,7-octatetrene	19.131	1134	1136.9	ND	ND	ND	ND	4.72
95	1,2,3,5-Tetramethtylbenzene	19.207	1127.9	1141.1	0.16	ND	ND	ND	ND
96	1,5,5,6-Tetramethylcyclohexa-1,3-diene	19.207	ND	1141.1	ND	ND	0.24	ND	ND
97	(E,Z)-Alloocimene	19.207	1131	1141.1	ND	ND	ND	2.50	ND
98	p-Mentha-1,3,8-triene	19.283	ND	1145.2	ND	ND	ND	1.42	ND
99	2-Bornene	21.33	ND	1254.6	ND	0.07	ND	ND	ND
100	3-Isopropylidene-6-methyl-1-cyclohexene	21.34	1088	1255.2	0.05	ND	ND	ND	ND
101	2,6,10,10-Tetramethyl-1-oxaspiro[4.5]dec-6-ene	22.672	1298	1336.5	ND	ND	ND	0.63	1.64
102	(-)-α-Cubebene	23.663	1351	1366.9	ND	ND	ND	2.91	9.60
103	α-Terpinene	23.939	1020	1379.5	ND	ND	ND	ND	1.06
104	3,8-p-Menthadiene	24.005	1071	1382.5	ND	ND	ND	0.25	ND
105	Terpinolene	24.101	1090	1386.9	ND	ND	0.29	ND	ND
106	Cyclohexene, 1-methyl-3- (1-methylethenyl)-	24.101	ND	1386.9	ND	ND	ND	2.15	ND
107	β-Elemene	24.662	1392	1410.6	ND	ND	2.14	ND	19.12
108	β-Caryophyllene	25.586	1419	1446.3	ND	1.18	5.23	17.41	34.36
109	(E)-α-Bergamotene	25.69	1450	1450.3	ND	ND	ND	0.24	1.96
110	(E)-β-Farnesene	25.957	1459	1460.6	ND	ND	ND	1.46	5.45
111	(E)-Germacrene D	26.328	1481	1474.9	ND	ND	ND	ND	0.81
112	α-Caryophyllene	26.49	1454	1481.2	0.30	0.86	3.59	ND	22.24
113	Naphthalene, 1,2,3,4,4a,5,6,7-octahydro-4a,8-dimethyl-2- (1-methylethenyl)-	27.271	1491.8	1511.7	ND	ND	6.77	27.01	96.67
114	β-Selinene	27.404	1475	1517.0	ND	ND	ND	ND	44.37
115	(R)-Valencene	27.509	1494	1521.2	ND	ND	ND	ND	17.73
116	Naphthalene, 1,2,3,7,8,8a-hexahydro-5,8a-dimethyl-3- (1-methylethenyl)-, (3R,8aR)-	27.557	ND	1523.1	ND	ND	3.24	9.83	0.20
117	γ-Cadinene	27.604	1502	1525.0	ND	ND	ND	ND	4.24
118	Eremophilene	27.661	1503	1527.2	ND	ND	ND	2.14	ND
119	1H-Cycloprop[e]azulene, decahydro-1,1,7-trimethyl-4-methylene-, (1aR,4aR,7R,7aR,7bS)-rel-	27.757	ND	1531.0	ND	ND	ND	1.26	1.37
120	(+)-Calarene	27.994	1432	1540.5	ND	ND	ND	ND	1.31
121	Δ-Cadinene	28.271	1525	1551.5	ND	ND	0.25	4.82	ND
122	Naphthalene, 1,2,3,4,4a,5,6,8a-octahydro-4a,8-dimethyl-2- (1-methylethenyl)-, (2S,4aR,8aR)-	28.366	1518	1555.3	ND	ND	ND	7.51	ND
123	Cadinadiene,cadina-1,4-diene	28.566	1542	1563.2	ND	ND	ND	0.22	ND
124	Elemicin	28.709	1554	1568.9	ND	ND	0.18	2.64	11.61
125	α-Calacorene	28.87	1540	1570.0	ND	ND	ND	0.15	1.34
126	Caryophyllene oxide	29.937	1617	1621.9	ND	ND	0.55	1.01	ND
127	α-Bulnesene	31.403	1526	1693.9	ND	ND	ND	0.71	ND
Alkenes (44)					0.56	5.00	56.54	143.01	389.34
128	Cyclohexane	7.183	ND	ND	0.26	0.12	ND	ND	ND
129	Methylcyclohexane	8.411	736	ND	ND	ND	ND	ND	0.70
130	(Z)-1,3-Dimethylcyclopentane	15.818	ND	977.4	ND	ND	ND	0.46	ND
131	Bicyclo2.2.1heptane, 7,7-dimethyl-2-methylene-	18.312	951	1093.0	ND	ND	ND	0.16	ND
132	2,4-Pentanediyl Diacetate	18.922	ND	1117.7	ND	ND	ND	0.68	8.62
133	1-Methylene-4- (1-methylvinyl)cyclohexane	22.12	1007	1295.9	ND	ND	ND	ND	2.38
134	Cyclopropane, 1,1-dimethyl-2- (3-methyl-1,3-butadien-1-yl)-	22.273	ND	1303.4	ND	ND	ND	1.80	ND
135	1-Methylenespiro[4.4]nonane	24.377	ND	1399.5	ND	ND	ND	2.79	ND
136	C15 H24,	26.947	1477	1498.8	ND	ND	ND	ND	1.96
Alkanes (9)					0.26	0.12	ND	5.89	13.66
137	Methyl eugenol	24.805	1404	1416.1	ND	ND	ND	11.47	ND
138	2,4-Di-tert-butylphenol	27.69	1519	1528.4	0.13	ND	ND	ND	ND
139	2,6-Di-tert-butylphenol	27.69	ND	1528.4	ND	0.09	ND	ND	ND
140	Butylated hydroxytoluene	27.899	1514	1536.7	ND	0.66	ND	ND	ND
Phenols (4)					0.13	0.75	ND	11.47	ND
141	2-Methylfuran	9.239	600	ND	ND	ND	ND	ND	0.19
142	α-Agarofuran	28.994	1543	1580.3	ND	0.52	1.10	2.80	6.10
Heterocycles (2)					ND	0.52	1.10	2.80	6.29
143	4-Allylanisole	20.607	1199	1208.9	ND	ND	ND	11.00	16.35
144	2H-1-Benzopyran, 3,4,4a,5,6,8a-hexahydro-2,5,5,8a-tetramethyl-	22.596	ND	1318.2	ND	ND	ND	0.61	ND
145	Edulan I	22.863	1315	1330.4	ND	ND	1.29	ND	8.59
Others (5)					ND	ND	1.29	11.61	24.94

Note: RT represents Retention Time; RI represents the Retention Index, and the RI was calculated by C_7_~C_40_ n-alkanes on the HP-5 MS Capillary GC Column (Length 60 m, inner diameter 0.25 mm, and film thickness 0.25 µm; Agilent, USA); LRI represents the Literature Retention Index, and the LRI was collected from NIST Chemistry WebBook database: webbook.nist.gov; I, II, III, IV, and V represent the different stages of fruit maturity; ND indicates not detected.

**Table 2:** ROAV values of VOCs in *R. roxburghii* fruit at different ripening stages.

Odor Descriptions	Class	Compounds	Odor Threshold (mg/kg)	ROAV				
				I	II	III	IV	V
Green	Aldehydes	2- (E)-Hexenal	0.0887	625.22	238.06	219.73	2363.27	207.05
		(E)-2-Octenal	0.003	1862.51	ND	1572.29	310.21	ND
	Alcohols	1-Heptanol	0.0054	ND	ND	ND	ND	390.45
		(Z)-2-Hexen-1-ol	0.3593	ND	ND	ND	ND	2.33
	Esters	(Z)-3-Hexenyl acetate	0.013	3544.18	16,836.82	10,973.98	2272.36	ND
		(Z)-3-Hexenyl butanoate	0.5	ND	ND	31.07	2.52	ND
	Ketones	Geranylacetone	0.06	19.92	21.61	54.07	ND	68.93
Fruit	Aldehydes	(E)-2-Pentenal	0.98	4.43	2.24	2.86	4.11	8.49
	Esters	Isoamyl acetate	0.00015	ND	ND	3077.42	4794.89	33,556.71
		Hexyl acetate	0.115	29.99	243.78	119.23	24.50	27.90
		Ethyl caprylate	0.0193	ND	20.01	21.35	1636.14	223.99
		Hexyl butyrate	0.203	ND	2.49	6.06	ND	ND
		2-Methylbutyl acetate	0.005	ND	ND	ND	132.12	336.56
		Ethyl Hexanoate	0.0022	ND	ND	ND	14,562.77	6978.21
		Methyl hexanoate	0.07	ND	ND	ND	ND	125.28
	Ketones	Nootkartone	0.28	ND	ND	ND	4.73	ND
Citrus	Esters	Caprylic acid methyl ester	0.2	ND	ND	2.32	2.98	177.04
	Alkenes	(Z)-β-Ocimene	0.034	ND	85.38	326.18	1581.58	ND
		4-Isopropenyltoluene	0.085	ND	ND	ND	ND	7.00
Grass	Aldehydes	Hexanal	0.005	16,225.60	8652.63	9827.86	35,421.65	13,307.02
	Alcohols	(3E)-3-Hexen-1-ol	0.11	ND	ND	461.03	ND	930.99
Fat	Aldehydes	Octanal	0.000587	ND	ND	35,446.06	16,981.53	98,386.72
		1-Nonanal	0.0011	11,982.85	17,551.64	70,171.81	51,494.71	93,581.44
		(E)-2-Nonenal	0.00019	3919.81	2052.53	4809.00	ND	ND
	Alcohols	1-Nonanol	0.002	653.29	1427.64	ND	ND	ND
		α-Terpineol	1.2	ND	ND	ND	2.49	ND
	Alkenes	p-Mentha-1,3,8-triene	0.015	ND	ND	ND	94.69	ND
Soap	Aldehydes	Decyl aldehyde	0.003	778.21	754.34	1348.23	ND	ND
		(E)-2-Heptenal	0.04	413.38	246.49	282.08	236.53	ND
	Ketones	2-Heptanone	0.14	2.65	ND	3.20	ND	5.10
Balsamic	Alcohols	1-Pentanol	0.1502	ND	ND	2.37	1.41	21.28
	Alkenes	Styrene	0.0036	12.91	ND	143.33	265.82	ND
Mint	Aldehydes	β-Cyclocitral	0.003	218.73	ND	ND	579.84	ND
Flower	Alcohols	1-Hexanol	0.0056	4706.37	4174.89	3924.12	1422.84	2572.79
		Geraniol	0.0011	ND	417.14	361.15	ND	ND
	Phenols	Methyl eugenol	0.775	ND	ND	ND	14.80	ND
	Ketones	β-Lonone	0.000007	85,552.07	ND	ND	183,215.46	1,095,920.27
Sweet	Alcohols	Phenethyl alcohol	0.56423	1.83	ND	ND	ND	ND
		Neraniol	0.68	ND	ND	ND	ND	1.91
	Alkenes	(E)-β-Ocimene	0.034	ND	ND	643.42	59.67	73.85
		Caryophylene oxide	0.41	ND	ND	1.35	2.45	ND
	Others	4-Allylanisole	0.0075	ND	ND	ND	ND	2180.27
Metal	Alcohols	1-Octanol	0.1258	26.45	44.60	70.81	124.62	ND
Wood	Ketones	α-Ionone	0.00378	ND	ND	ND	131.65	2452.49
	Alkenes	α-Caryophyllene	0.16	1.87	5.36	22.47	ND	139.00
		β-Caryophyllene	0.064	ND	18.40	81.77	272.01	536.81
Caramel	Aldehydes	Benzaldehyde	0.75089	1.97	ND	ND	ND	3.01
	Ketones	4-Methoxy-2,5-dimethyl-3 (2H)-furanone	0.16	ND	ND	10.36	31.03	314.81
Mushroom	Ketones	6-Methyl-5-hepten-2-one	0.068	ND	42.48	95.68	39.79	104.31
Pungent	Ketones	Ethyl vinyl ketone	0.023	ND	ND	280.88	ND	891.90
Sweat	Acids	Octanoic acid	3	ND	ND	4.08	3.28	89.64
		Hexanoic acid	5	ND	ND	ND	ND	1.59
Lemon	Alkenes	α-Terpinene	0.04	ND	ND	ND	ND	13.23

Note: Odor descriptions are sourced from Flavornet by Terry Acree and Heinrich Arn (https://www.flavornet.org, accessed on 15 April 2024) and the Flavor Ingredient Library (https://www.femaflavor.org/flavor-library, accessed on 15 April 2024). The odor threshold refers to the concentration of substances in water as detailed in the database. I, II, III, IV, and V represent the different stages of fruit maturity. ND indicates not detected.

## Data Availability

The original contributions presented in this study are included in this article/Supplementary Material, further inquiries can be directed to the corresponding author.

## Supplementary Materials

The following supporting information can be downloaded at: https://www.mdpi.com/article/10.3390/foods13182893/s1, Figure S1: Clustering heat map of VOCs in *R. roxburghii* fruit at different ripening stages. Figure S2. VIP diagram of VOCs. Red bars represent the VOCs with VIP value over 1.
