# Supplementary material for: Dynamic Changes of Active Components and Volatile Organic Compounds in Rosa roxburghii Fruit during the Process of Maturity
Source: Foods. 2024 Sep 12;13(18):2893. doi: 10.3390/foods13182893 (PMC11431035; doi:10.3390/foods13182893)
Supplement: Supplementary file 1 [file foods-13-02893-s001.zip › foods-3189951-supplementary.pdf]

Supplementary Material

Dynamic Changes of Active Components and Volatile Organic Compounds in *Rosa roxburghii* Fruit during the Process of Maturity

Su Xu, Junyi Deng, Siyao Wu, Qiang Fei, Dong Lin, Haijiang Chen, Guangcan Tao, Lingshuai Meng, Yan Hu and Fengwei Ma *

College of Food Science and Engineering, Guizhou Engineering Research Center for Characteristic Flavor Perception and Quality Control of Dual-Food Homologous Resources, Guiyang University, Guiyang 550005, China; xs8515@126.com (S.X.); 13187337873@163.com (J.D.); ceci41@126.com (S.W.); fqorganic@163.com (Q.F.); gyulindong@163.com (D.L.); b05chenhj@126.com (H.C.); tgcan@126.com (G.T.); 15040260380@163.com (L.M.); huyanswu@163.com (Y.H.)

* Correspondence: sp0024@gyu.edu.cn

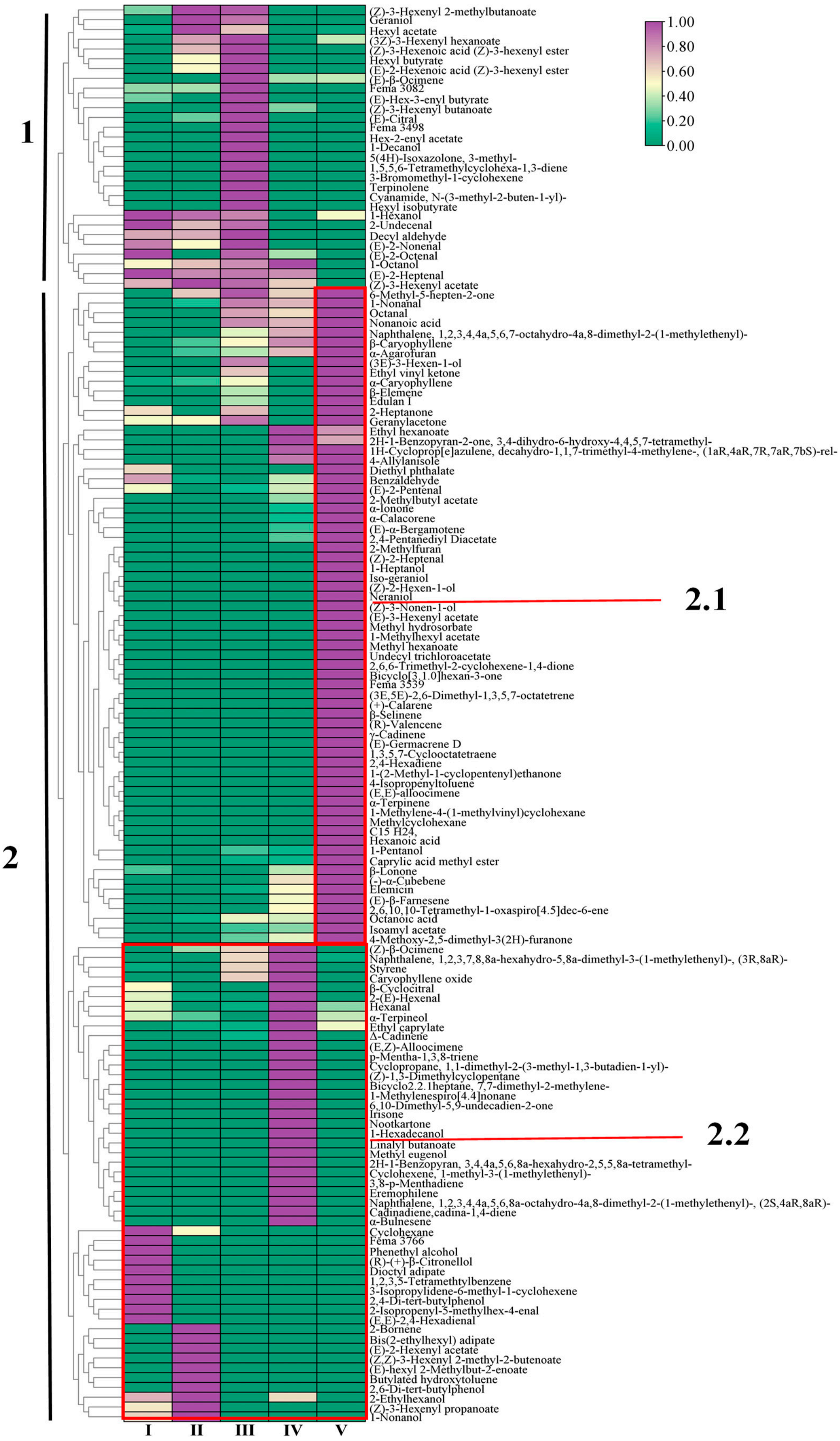

Figure S1. Clustering heat map of VOCs in *R. roxburghii* fruit at different ripening stages.

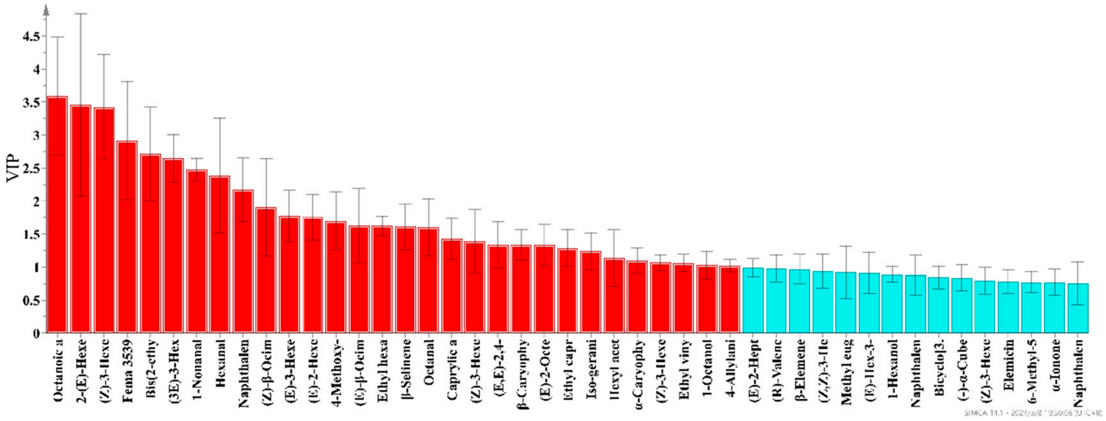

Figure S2. VIP diagram of VOCs. Red bars represent the VOCs with VIP value over 1.
